# U‐Shaped Relationship Between CT‐Measured Liver‐To‐Spleen Volume Ratio and Mortality in HBV‐ACLF Patients

**DOI:** 10.1111/jvh.70076

**Published:** 2025-09-16

**Authors:** Libo Yan, Man Yuan, Mao Su, Kunping Cui, Xiangnan Teng, Fang Yuan, Lang Bai

**Affiliations:** ^1^ Center of Infectious Diseases West China Hospital of Sichuan University Chengdu China; ^2^ Department of Radiology, West China Hospital Sichuan University Chengdu China

**Keywords:** acute‐on‐chronic liver failure, hepatitis B virus, liver‐to‐spleen volume ratio, U‐shaped

## Abstract

Hepatitis B virus‐related acute‐on‐chronic liver failure (HBV‐ACLF) is a life‐threatening condition with high short‐term mortality, making early prognosis crucial. The liver‐to‐spleen volume ratio (LSVR) provides important prognostic information but is not included in current tools. This study evaluated the link between LSVR from computed tomography and short‐term mortality in HBV‐ACLF patients. The study included 278 patients, divided into five groups based on LSVR quintiles. The main outcome was 28‐day mortality, with a secondary focus on 90‐day mortality. Multivariable Cox regression and restricted cubic splines were used to analyse the LSVR‐mortality relationship. Participants had a mean age of 48 years, 82.7% were male, with 28‐ and 90‐day mortality rates of 23.4% and 31.3%, respectively. After adjusting for covariates, the risk of 28‐day mortality was elevated by 553% (OR 6.53, 95% CI 1.86–23) in the highest quintile of LSVR (Q5 ≥ 3.6) and by 343% (OR 4.43, 95% CI 1.14–17.16) in the lowest quintile (Q1 ≤ 1.6), as compared to the reference quintile (Q3 2.4–2.9). The curve‐fitting results showed a U‐shaped relationship between LSVR and the risk of 28‐day mortality and 90‐day mortality, with an infection point of 2.7. There is a U‐shaped relationship between LSVR and mortality in HBV‐ACLF patients. Higher or lower LSVR is associated with an increased risk of short‐term mortality in HBV‐ACLF patients.

## 1 Introduction

Acute‐on‐chronic liver failure (ACLF) has been recognised as a complex syndrome that is associated with a high short‐term mortality rate. The only definitive and effective treatment is liver transplantation, which could not be widely used due to the lack of donor livers and the high cost in China. Early diagnosis and early determination of the prognosis are critical to guide clinical management and decrease the high mortality rate. Chronic hepatitis B virus (HBV) infection is the primary aetiology of ACLF in China, accounting for over 60% of all cases [1]. The traditional scoring systems for HBV‐related ACLF (HBV‐ACLF), such as the model for end‐stage liver disease (MELD) score, the MELD‐sodium (MELD‐Na) score, the Child‐Pugh score (CPS) and the Indocyanine green clearance (ICG) test, have limited accuracy for HBV‐ACLF prognosis [2]. Given the high incidence and morbidity rates, building a comprehensive algorithm to evaluate the patient is essential. The development of a more accurate predictive tool for MELD‐ACLF prognosis has been the research hotspot in the last decade.

Medical imaging modalities such as computed tomography (CT) are noninvasive and can be easily performed several times during the clinical course. Liver volume (LV) could reflect the balance of hepatic injury from the morphology perspective, resulting in hepatocyte necrosis and structural collapse with that of hepatic regeneration [3]. Smaller LV indicated more severe damage and necrosis of liver failure [4, 5, 6]. It might also parallel the compromise of hepatic metabolic and synthetic function. Spleen volume (SV) reflects liver cirrhosis and portal hypertension [7, 8]. In clinical practice, it is often observed that ACLF patients with significantly reduced liver volume and splenomegaly have poor prognosis. The estimation of LV by CT is widely used for assessing the feasibility of tumour resection or living donor transplantation [9, 10, 11, 12, 13]. When chronic liver disease suffers an acute insult, massive necrosis, shrinkage and structural collapse occur in liver tissue, leading to volume decrease with hepatic reserve dysfunction [14]. However, to what extent these morphological changes could be related to key events in HBV‐ACLF has not been fully addressed.

Because the volumes of the liver and the spleen obviously differ at different pathological stages of liver failure, we postulated that the ratio of the volume of the liver to that of the spleen might have clinical significance. Therefore, we conducted a retrospective cohort study using the HBV‐ACLF database in West China Hospital to investigate the CT‐derived liver‐to‐spleen volume ratio (LSVR) and assess the LSVR prognostic importance in HBV‐ACLF.

## 2 Materials and Methods

### 2.1 Study Design

This retrospective study included patients admitted with HBV‐ACLF to the West China Hospital of Sichuan University from February 2018 to September 2023. Clinical data from all selected patients were reviewed, including demographic features, predisposing factors, clinical manifestations, results of laboratory tests, CT images, treatments and prognosis. The Ethics Committee of the West China Hospital of Sichuan University (Chengdu, Sichuan, China) approved the study protocol following the ethical guidelines of the 1975 Declaration of Helsinki (approval number: 2023–1755). Informed consent was not obtained since it was a retrospective analysis of the collected data, and the participants' identities were kept confidential.

ACLF was defined according to the following criteria specified by the Asian Pacific Association for the Study of the Liver [15] and the Guideline for Diagnosis and Treatment of Liver Failure in China [2]: (1) extreme fatigue with severe digestive symptoms such as apparent anorexia, abdominal distension, or nausea and vomiting; (2) the acute deterioration of pre‐existing chronic liver disease/cirrhosis; (3) serum total bilirubin (TBiL) ≥ 178 μmol/L; (4) coagulopathy (INR ≥ 1.5). Subjects with other clinical liver diseases, such as autoimmune liver diseases, alcoholic liver disease, drug‐induced liver injury, hepatocellular carcinoma, hematologic malignancy, bile duct obstruction, Wilson's disease, chronic hepatitis C infection, or human immunodeficiency virus coinfection, were excluded.

**FIGURE 1:**
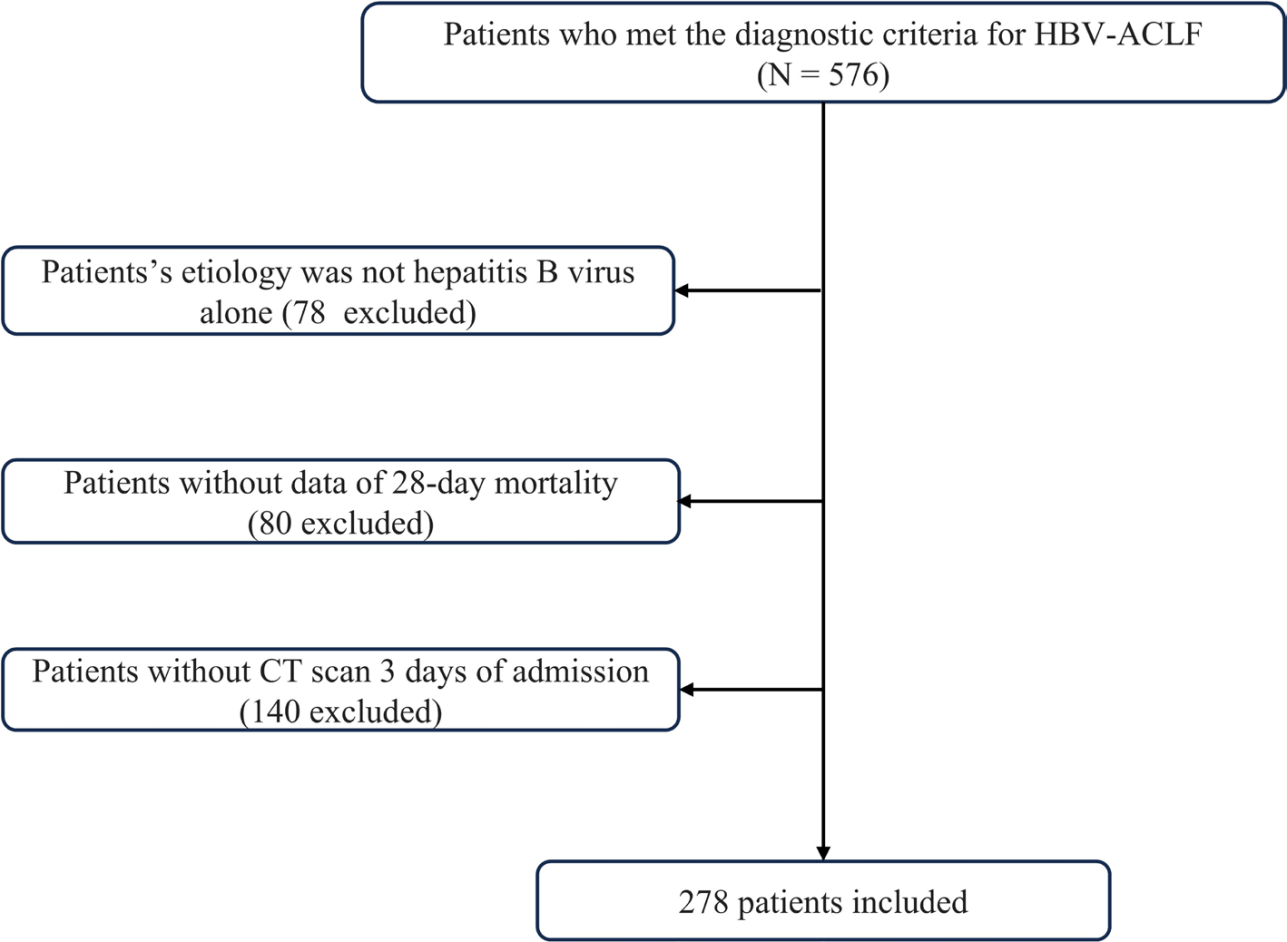
Flowchart of study patients.

A total of 576 patients who met the diagnostic criteria for HBV‐ACLF and were firstly admitted to the West China Hospital of Sichuan University were included. After excluding patients whose aetiology was not hepatitis B alone, without data on 28‐day mortality, and without a CT scan 3 days after admission, 278 participants were enrolled in the study (Figure 1).

### 2.2 Main Variables and Outcome Variables

**FIGURE 2:**
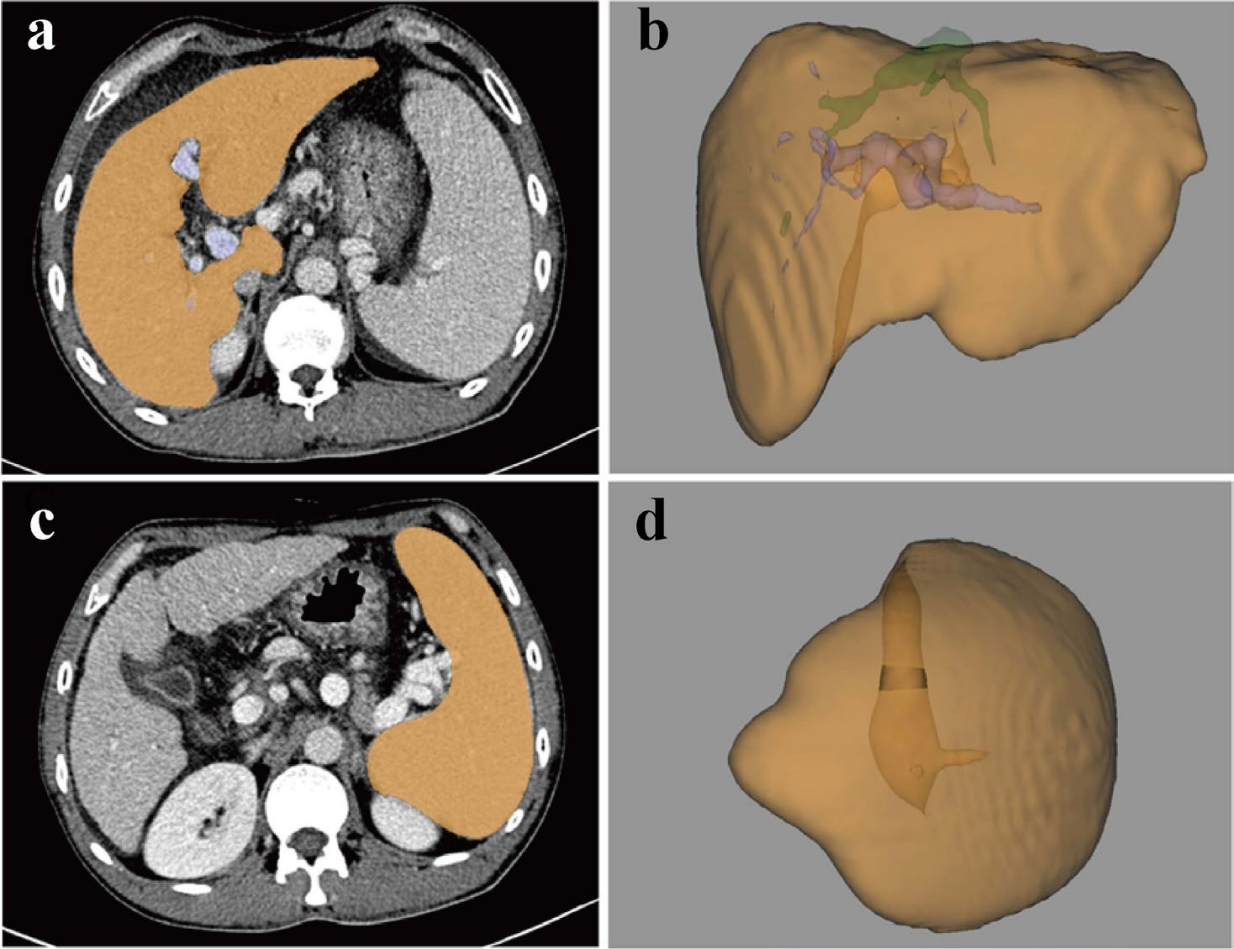
Illustrative liver and spleen volumes. A 46‐year‐old man with HBV‐ACLF and decompensated liver cirrhosis. ALT: 373 U/L, AST: 315 U/L, TBiL: 427.6 μmol/L, INR: 1.76, albumin: 28 g/L, LV: 1356.7 cm^3^, SV: 855.7 cm^3^, LSVR: 1.6. (a) Liver label. (b) 3D reconstruction of liver images. (c) Spleen label. (d) 3D reconstruction of spleen images.

The primary variable of the study was CT‐derived LSVR at the time of admission. CT images were acquired using scanners from multiple manufacturers. Scanning was performed with the patient holding their breath at the end of inspiration. Non‐contrast CT images were first obtained, followed by the intravenous administration of 120 to 130 mL of contrast agent at a flow rate of 3 mL/s using a mechanical injector. Triphasic contrast‐enhanced CT scans were then performed during the arterial, portal venous and delayed phases. Portal venous phase images were utilised for segmentation. LV and SV were measured for each subject using IntelliSpace Portal software (Philips, Amsterdam, Netherlands). LV was segmented automatically, whereas SV was segmented semi‐automatically by manually selecting three points around the spleen. Subsequently, liver and spleen labels, as well as 3D reconstruction images and volume data, were generated and visualised (Figure 2). We acquired LV and SV from CT images within 3 days of admission. LV and SV were determined by a hepatic radiologist who was unaware of patients' outcomes and clinical characteristics. LSVR was calculated by the formula: LV/SV.

The primary outcome of the study was 28‐day mortality after admission. In addition to this, we analysed the relationship between CT‐derived LSVR and 90‐day mortality.

### 2.3 Statistical Analysis

This study aimed to determine the association between LSVR and 28‐day mortality in patients with HBV‐ACLF. Based on the quintile of the LSVR, the study population was classified into five groups (Q1: ≤ 1.6, Q2: 1.7–2.3, Q3: 2.4–2.9, Q4: 3.0–3.5, Q5: ≥ 3.6). Continuous data with a skewed distribution were expressed as medians and interquartile ranges. Kruskal–Wallis tests were used for comparisons between groups. Categorical variables were expressed as proportions (%). The chi‐square test or Fisher's exact test were used for comparison between groups. Cox proportional hazard regression models were performed to calculate hazard ratios and 95% confidence intervals (CIs) for LSVR and 28‐day mortality in patients with HBV‐ACLF. Crude model analysis adjusted for none. Model 1 was adjusted for age and sex. Model 2 was adjusted for model 1 plus hypertension and diabetes mellitus. Model 3 was adjusted for model 2 plus variables with an effect value > 10% (body temperature, white blood cell (WBC), platelets, TBiL and sodium). Restricted cubic spline (RCS) analysis was performed to examine the non‐linearity of LSVR and 28‐day mortality. Four knots (5th, 35th, 65th and 95th percentiles of LSVR distribution) were used for RCS modelling, with the lowest level of risk as the reference value. If a non‐linear correlation was detected, the two‐piecewise was used to calculate the inflection point between LSVR and 28‐day mortality and 90‐day mortality after adjusting for the variables in model 3. All analyses were performed using R Statistical Software (version 4.2.2, http://www.r‐project.org) and the Free Statistics analysis platform (Version 1.8). A two‐tailed test was performed, and a *p* value of < 0.05 was considered significant.

## 3 Results

### 3.1 Baseline Characteristics

Table 1 outlines the baseline characteristics of participants grouped by LSVR levels. The mean age of all participants was 48 (41, 54) years, of which 82.7% were male. There were 95.3% of participants with cirrhosis, 6.5% of participants with hypertension, 9.0% with diabetes, 11.9% with gastrointestinal bleeding and 57.2% with infection. The 28‐day mortality rate was 23.4%, and the 90‐day mortality rate was 31.3%. Patients with higher LSVR had higher levels of WBC, platelets (PLT) and aspartate aminotransferase.

**TABLE 1:** Baseline characteristics of participants according to LSVR.

Characteristics	Total (*N* = 278)	LSVR					*p*
		Q1 (≤ 1.6) *N* = 51	Q2 (1.7–2.3) *N* = 56	Q3 (2.4–2.9) *N* = 52	Q4 (3.0–3.5) *N* = 61	Q5 (≥ 3.6) *N* = 58	
Age (year)	48.0 (41.0, 54.0)	46.0 (39.0, 51.0)	45.0 (38.8, 53.5)	46.5 (42.5, 52.2)	50.0 (44.0, 55.0)	51.0 (47.0, 61.8)	0.004
Gender (male %)	230 (82.7)	44 (86.3)	48 (85.7)	41 (78.8)	51 (83.6)	46 (79.3)	0.763
Cirrhosis (%)	265 (95.3)	51 (100)	55 (98.2)	44 (84.6)	58 (95.1)	57 (98.3)	0.002^a^
Hypertension (%)	18 (6.5)	2 (3.9)	1 (1.8)	1 (1.9)	4 (6.6)	10 (17.2)	0.006^a^
Diabetes (%)	25 (9.0)	4 (7.8)	4 (7.1)	4 (7.7)	6 (9.8)	7 (12.1)	0.907^a^
Gastrointestinal bleeding (%)	33 (11.9)	10 (19.6)	8 (14.3)	5 (9.6)	5 (8.2)	5 (8.6)	0.302
Infection (%)	159 (57.2)	33 (64.7)	32 (57.1)	27 (51.9)	29 (47.5)	38 (65.5)	0.220
Body temperature (°C)	36.5 (36.4, 36.7)	36.5 (36.5, 36.8)	36.6 (36.5, 36.9)	36.5 (36.5, 36.6)	36.6 (36.5, 36.7)	36.5 (36.3, 36.7)	0.165
Bio‐characteristics							
WBC (×10^9^/L)	6.2 (4.6, 8.8)	5.2 (3.4, 7.3)	5.8 (4.3, 6.6)	6.1 (5.0, 7.2)	7.0 (5.0, 10.6)	7.6 (6.0, 10.2)	< 0.001
PLT (×10^9^/L)	81.0 (56.2, 116.8)	46.0 (31.0, 70.0)	74.5 (59.5, 99.2)	93.5 (64.0, 124.0)	95.0 (68.0, 128.0)	113.5 (71.5, 137.8)	< 0.001
Haemoglobin (g/L)	122.0 (106.0, 137.0)	112.0 (80.0, 132.0)	121.5 (101.8, 137.2)	125.0 (115.2, 133.0)	128.0 (116.0, 139.0)	124.5 (109.0, 136.8)	0.020
ALT (U/L)	174.5 (63.8, 562.0)	81.0 (31.5, 195.5)	182.5 (65.2, 735.2)	346.5 (95.8, 743.2)	181.0 (74.0, 533.0)	224.0 (90.2, 629.5)	< 0.001
AST (U/L)	165.0 (80.5, 381.0)	87.0 (59.0, 193.5)	169.0 (78.8, 513.8)	204.5 (113.8, 554.2)	170.0 (76.0, 347.0)	221.0 (111.5, 430.8)	< 0.001
TBiL (μmol/L)	307.9 (214.1, 422.8)	325.0 (197.6, 445.6)	326.6 (233.1, 428.2)	275.9 (197.6, 387.9)	310.9 (246.8, 415.9)	301.6 (200.8, 371.6)	0.370
Albumin (g/L)	32.4 (29.2, 35.9)	31.8 (29.0, 36.1)	33.3 (30.6, 38.4)	31.8 (29.0, 35.2)	33.3 (29.3, 35.9)	32.2 (29.2, 34.7)	0.333
Creatinine (μmol/L)	76.0 (61.2, 90.0)	76.0 (65.5, 99.0)	72.0 (60.8, 85.0)	76.0 (64.5, 83.5)	77.0 (58.0, 88.0)	78.5 (64.8, 92.0)	0.218
Potassium (mmol/L)	3.7 (3.4, 4.1)	3.8 (3.4, 4.1)	3.7 (3.4, 4.0)	3.6 (3.4, 4.0)	3.8 (3.5, 4.1)	3.9 (3.5, 4.2)	0.531
Sodium (mmol/L)	135.2 (132.8, 138.0)	134.9 (131.1, 137.2)	135.3 (133.3, 139.1)	135.8 (132.6, 138.6)	135.3 (132.3, 137.4)	135.2 (133.4, 137.7)	0.617
Prothrombin time (s)	21.7 (18.6, 26.4)	21.8 (17.8, 26.6)	21.3 (19.0, 26.9)	21.4 (19.0, 25.2)	22.7 (19.3, 25.5)	21.1 (16.8, 26.5)	0.640
INR	2.0 (1.7, 2.4)	2.0 (1.6, 2.4)	1.9 (1.7, 2.4)	1.9 (1.7, 2.2)	2.0 (1.7, 2.4)	1.9 (1.5, 2.2)	0.570
Imaging characteristics							
Liver volume (cm^3^)	1032.2 (871.0, 1269.4)	927.4 (756.0, 1152.9)	1015.2 (913.0, 1216.6)	1121.3 (897.4, 1348.0)	1047.9 (880.3, 1218.6)	1099.5 (892.5, 1364.9)	0.058
Spleen volume (cm^3^)	405.5 (297.3, 582.2)	806.1 (673.7, 967.1)	521.4 (453.5, 604.3)	428.0 (341.5, 525.8)	318.2 (270.1, 372.0)	237.2 (179.3, 304.8)	< 0.001
Outcomes							
28‐day mortality	65 (23.4)	15 (29.4)	10 (17.9)	5 (9.6)	10 (16.4)	25 (43.1)	< 0.001
90‐day mortality	87 (31.3)	17 (33.3)	13 (23.2)	9 (17.3)	19 (31.1)	29 (50)	0.003

Abbreviations: ALT, alanine transaminase; AST, aspartate aminotransferase; INR, international normalised ratio; LSVR, liver‐to‐spleen volume ratio; PLT platelets; TBiL, total bilirubin; WBC, white blood cell.

^a^ Fisher's exact test.

### 3.2 Multivariable Cox Regression Analysis

After performing multicollinearity analysis, we constructed three multivariable Cox regression models to assess the relationship between LSVR and the 28‐day mortality risk in patients. Table 2 details the relationship between LSVR and the 28‐day mortality risk, with effect values expressed as odds ratios and 95% CIs. In the unadjusted model, the risk of mortality was increased by 292% (OR 3.92, 95% CI 1.3–11.78) in Q1 when compared with LSVR in Q3, and the risk of mortality was elevated by 612% (OR 7.12, 95% CI 2.47–20.52) in the highest quintile Q5. Additionally, in model 3 (adjusted for age, sex, hypertension, diabetes mellitus, body temperature, WBC, PLT, TBiL and sodium), using Q3 as the reference, the risk of mortality was elevated by 343% (OR 4.43, 95% CI 1.14–17.16) in Q1 and 553% (OR 6.53, 95% CI 1.86–23) in Q5, respectively. The risk of mortality was lower at LSVR of 2.4 to 2.9.

**TABLE 2:** Association of LSVR with 28‐day mortality in patients with HBV‐ACLF.

Variable	Crude model		Model 1		Model 2		Model 3	
	OR 95% CI	*p*	OR 95% CI	*p*	OR 95% CI	*p*	OR 95% CI	*p*
Q1 (≤ 1.6)	3.92 (1.3–11.78)	0.015	4.45 (1.44–13.76)	0.010	4.29 (1.39–13.26)	0.011	4.43 (1.14–17.16)	0.031
Q2 (1.7–2.3)	2.04 (0.65–6.44)	0.222	2.11 (0.65–6.83)	0.213	2.09 (0.65–6.78)	0.219	2.23 (0.59–8.46)	0.238
Q3 (2.4–2.9)	1 (Ref)		1 (Ref)		1 (Ref)		1 (Ref)	
Q4 (3.0–3.5)	1.84 (0.59–5.79)	0.295	1.68 (0.52–5.41)	0.382	1.56 (0.48–5.07)	0.457	1.38 (0.36–5.26)	0.635
Q5 (≥ 3.6)	7.12 (2.47–20.52)	< 0.001	5.87 (1.97–17.46)	0.001	5.07 (1.69–15.27)	0.004	6.53 (1.86–23)	0.003
*p* for trend		0.003		0.025		0.063		0.038

*Note:* Crude model adjusted for none. Model 1 adjusted for age, sex. Model 2 adjusted for Model 1 + hypertension and diabetes mellitus. Model 3 adjusted for Model 2 + body temperature, white blood cell, platelets, total bilirubin and sodium.

Abbreviations: CI, confidence interval; HBV‐ACLF, HBV‐related acute‐on‐chronic liver failure; LSVR, liver‐to‐spleen volume ratio; OR, odds ratio; Ref, reference.

### 3.3 Analyses of the U Shape Association

**FIGURE 3:**
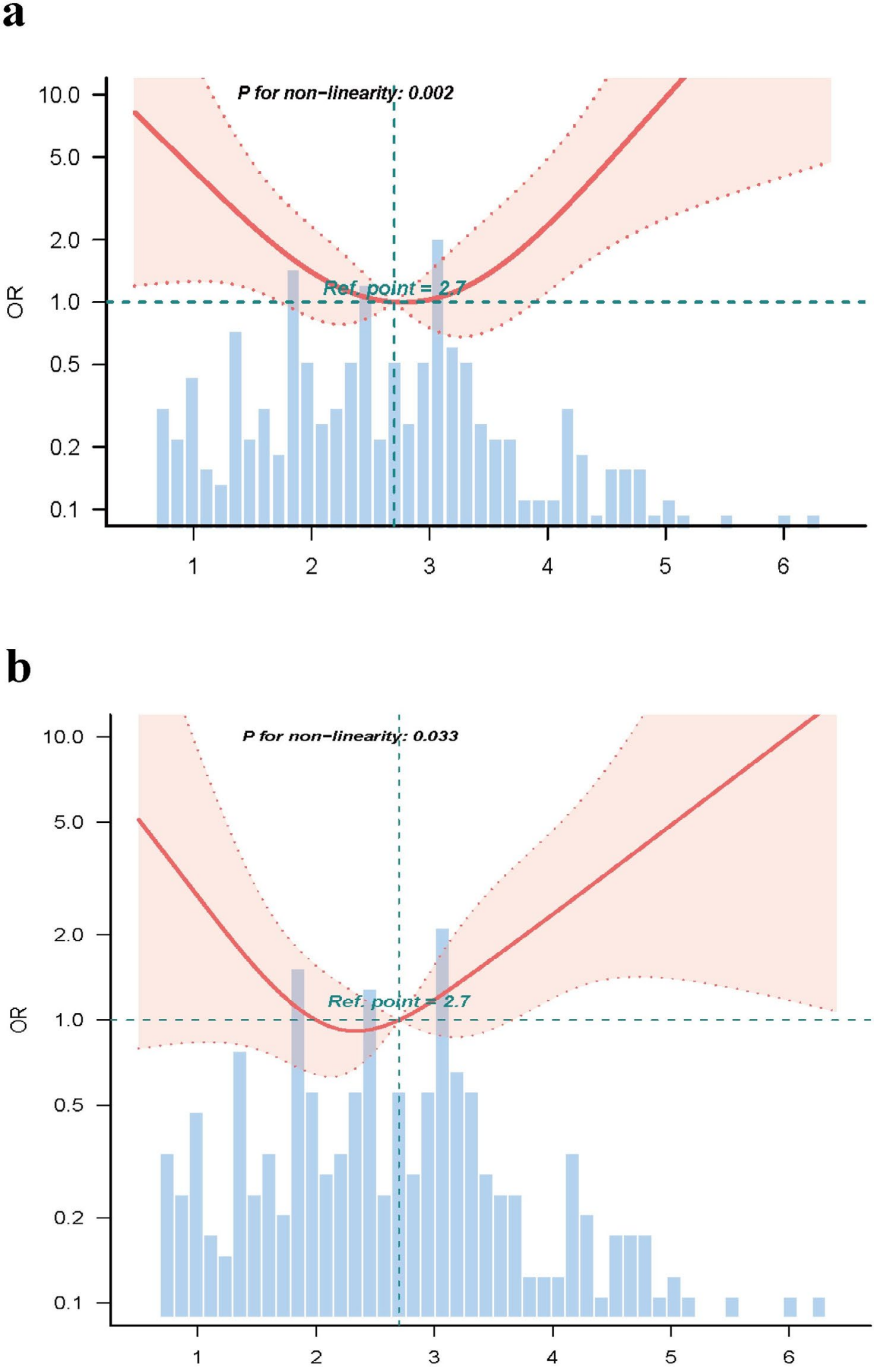
Relationship between liver‐to‐spleen volume ratio and short‐term mortality. (a) 28‐day mortality. (b) 90‐day mortality. Curves represent estimated adjusted odds ratios, and shaded ribbons represent 95% confidence intervals. The horizontal dashed line represents an odds ratio of 1.0. Ninety‐eight per cent of the data is displayed. OR, odds ratio; Ref, reference.

After adjusting for covariates in model 3, we found a U‐shaped relationship between LSVR and 28‐day mortality in curve fitting (*p* for non‐linearity = 0.002, Figure 3a). We found an infection point of LSVR at 2.7 using the RCS. When LSVR ≥ 2.7 (Table 3), the 28‐day mortality risk increased by 182.2% (OR 2.822, 95% CI 1.452–5.482) for every one increase. At LSVR < 2.7, the 28‐day mortality risk decreased by 83.2% for every one increase in LSVR. We also found this U‐shaped relationship between LSVR and 90‐day mortality risk (*p* for non‐linearity = 0.033, Figure 3b). At LSVR ≥ 2.7 (Table 4), there was a 90% (OR 1.900, 95% CI 0.993–3.638) increase in the 90‐day mortality risk for every one increase. In contrast, the 90‐day risk of mortality decreased by 74.4% for every one increase in LSVR when LSVR < 2.7.

**TABLE 3:** Threshold effect analysis of the relationship between LSVR and 28‐day mortality of patients with HBV‐ACLF.

Threshold of LSVR	OR	95% CI	*p*
< 2.7	0.168	0.042–0.675	0.012
≥ 2.7	2.822	1.452–5.482	0.0022
Nonlinear test			< 0.001

*Note:* The data were adjusted for model 3.

Abbreviations: CI, confidence interval; HBV‐ACLF, HBV‐related acute‐on‐chronic liver failure; LSVR, liver‐to‐spleen volume ratio; OR, odds ratio; Ref, reference.

**TABLE 4:** Threshold effect analysis of the relationship between LSVR and 90‐day mortality of patients with HBV‐ACLF.

Threshold of LSVR	OR	95% CI	*p*
< 2.7	0.256	0.082–0.801	0.0192
≥ 2.7	1.900	0.993–3.638	0.0526
Nonlinear test			0.005

Abbreviations: CI, confidence interval; HBV‐ACLF, HBV‐related acute‐on‐chronic liver failure; LSVR, liver‐to‐spleen volume ratio; OR, odds ratio; Ref, reference.

## 4 Discussion

The present study investigated the relationship between LSVR and short‐term mortality risk in HBV‐ACLF patients. After adjusting for relevant confounders, we found a U‐shaped relationship between LSVR and short‐term mortality risk at 28 and 90 days in HBV‐ACLF patients, respectively. Lower or higher LSVR is associated with an increased risk of short‐term mortality, with an infection point of 2.7. The risk of mortality was lower at LSVR of 2.4 to 2.9. LSVR should be of interest in identifying HBV‐ACLF patients at high risk of short‐term mortality because of its ease and accessibility. To our knowledge, the LVSR prognostic role in HBV‐ACLF patients has not been reported.

The morphology of the liver lobe and spleen changes with the progression of cirrhosis, and the changes in liver lobe volume may result from the special anatomy of the portal vein. The LV and SV can be used to assess the severity of portal hypertension and cirrhosis. LSVR may capture cirrhosis‐related changes reflected in both liver and spleen volumes as a composite index. A few previous studies have demonstrated that the LSVR based on CT is helpful for the detection of clinically significant portal hypertension and decompensated cirrhosis. Shi‐ping Yan et al. constructed a predictive equation combined with the LVSR and classification of varices to predict hepatic venous pressure gradient (HVPG) greater than 12 mmHg in hepatitis B patients with cirrhosis [16]. The area under the curve of this predictive equation was 0.919, and the sensitivity, specificity, positive predictive value and negative predictive value were 92.9%, 87.0%, 89.7% and 90.9%, respectively [16]. In addition, another study by Mario Romero‐Cristóbal et al. on cirrhosis patients who underwent liver transplantation or hepatocellular carcinoma resection revealed that the LVSR was significantly lower in decompensated patients [17]. However, regarding the prognostic role of LSVR, only two studies reported a correlation between LSVR and the survival of patients with cirrhosis. Yosuke Murata et al. conducted a study to evaluate the LVSR prognostic outcome in patients with primary biliary cirrhosis. They found that the prognosis was significantly poorer in the low LVSR (< 6.5) than in the high LVSR (≥ 6.5) group according to the Kaplan–Meier analysis (*p* = 0.0008) [18]. Meanwhile, Ji Hye Kwon included patients with HBV‐compensated cirrhosis, showing that patients with an LSVR of < 2.9 had significantly higher 3‐year risks of hepatic decompensation (16.7% vs. 2.5%, *p* < 0.001) and liver‐related death or transplantation (10.0% vs. 1.1%, *p* < 0.001) than those with an LSVR ≥ 2.9 [19]. When patients were stratified according to CPS (Child‐Pugh A vs. B–C) and MELD (< 10 vs. ≥ 10), an LSVR of < 2.9 was still associated with a higher risk of liver‐related events than an LSVR of ≥ 2.9 for all Child‐Pugh (*p* ≤ 0.045) and MELD (*p* ≤ 0.009) stratifications [19]. From these findings, it is natural to consider that the prognosis of cirrhosis is poorer in the low LVSR. In our study, there were 95.3% of HBV‐ACLF patients with cirrhosis. Thus, we focused on LVSR, which may reflect the prognosis of HBV‐ACLF as its risk factor. However, we found that a lower or higher LSVR is associated with an increased risk of mortality. These findings are inconsistent with the results of previous studies in cirrhosis patients. We speculated that this difference might depend on the acute liver failure of the included patients with severe damage and necrosis of hepatocytes in a short time. The LSVR ratio depended mainly on alterations in SV because LV did not differ significantly between the five groups in our study.

Currently, various parameters are preoperatively used as indicators of liver prognostic factors, such as MELD score, MELD‐Na score, CPS, ICG test and serum‐based markers [20]. However, the measurement of several serum‐based markers may not always be possible to perform in every institution. Besides, it is difficult to determine accurately due to the wide variety of complex functions, including protein synthesis as well as metabolic, immune and storage functions. In addition, the MELD score and CPS were based on the European population, among which alcoholic and fatty liver diseases were the most dominant aetiologies. In contrast, LVSR is the volumetric index that best captures this pathophysiological process, which can easily and simply be measured by performing quick, semi‐automatic post‐processing of the already obtained images from routine preoperative CT scans without requiring additional tests. In clinical practice, since easy, reproducible and noninvasive tools that can be performed without any specific skills are preferable to predict valuable add‐on information, LVSR may potentially be useful in many institutions and not only in high‐volume centres.

Our study has the following advantages. First, we are the first to study the relationship between LSVR and mortality risk in HBV‐ACLF patients. Second, we found a U‐shaped relationship between LSVR and mortality risk at 28 and 90 days using curve fitting. Some disadvantages have to be considered. First, the subjects in our study are the patients with HBV‐ACLF. When compared with other types of liver failure, such as alcoholic, autoimmune and drug‐induced liver failure, the results may be different. As reported [21], in patients with acute liver injury or failure, LV shows marked variation by the cause of disease and in prognostic importance. Second, this was a retrospective study unable to make causal inferences, but we adjusted for potential confounders to ensure the stability of the results. Third, this is a single‐centre study; future multi‐centre prospective studies are needed to validate this conclusion.

Overall, the findings of our study suggested there existed an inverted U‐shaped relationship between LSVR and the risk of short‐term mortality in HBV‐ACLF patients. Lower or higher LSVR is associated with an increased risk of short‐term mortality, with an infection point of 2.7. It demonstrated LSVR as a prognostic marker of short‐term mortality in HBV‐ACLF patients. These volumetric indices can be easily obtained and reproduced by CT scan and provide relevant prognostic information in HBV‐ACLF patients. Further research is needed to establish short‐term mortality and explore the role of LSVR in this relationship.

## Author Contributions

Libo Yan carried out the design of the study and manuscript writing. Man Yuan was involved in manuscript writing. Mao Su and Kunping Cui carried out data analysis. Xiangnan Teng carried out data collection. Fang Yuan and Lang Bai carried out the design of the study and manuscript editing. All authors have read and agreed to the published version of the manuscript.

## Conflicts of Interest

The authors declare no conflicts of interest.

## Data Availability

The data that support the findings of this study are available on request from the corresponding author. The data are not publicly available due to privacy or ethical restrictions.
